# The COVID-19 Outbreak May Be Associated to a Reduced Level of Care for Breast Cancer. A Comparative Study with the Pre-COVID Era in an Italian Breast Unit

**DOI:** 10.3390/healthcare8040474

**Published:** 2020-11-11

**Authors:** Alessandro Fancellu, Valeria Sanna, Corrado Rubino, Maria Laura Ariu, Claudia Piredda, Gian Quirico Piana, Pietrina Cottu, Angela Spanu, Antonio Cossu, Giulia Deiana, Alberto Porcu

**Affiliations:** 1 Unit of General Surgery 2—Clinica Chirurgica, Department of Medical, Surgical and Experimental Sciences, University of Sassari, 07100 Sassari, Italy; mlaura.ariu@gmail.com (M.L.A.); claudiapi53@gmail.com (C.P.); icopiagqr@gmail.com (G.Q.P.); pcottu@yahoo.it (P.C.); giulia.deiana2@gmail.com (G.D.); alberto@uniss.it (A.P.); 2 Department of Oncology, AOU Sassari, Via E. De Nicola, 07100 Sassari, Italy; valeria.sanna@aousassari.it; 3 Unit of Plastic Surgery, Department of Medical, Surgical and Experimental Sciences, University of Sassari, 07100 Sassari, Italy; corrado.rubino@aousassari.it; 4 Unit of Nuclear Medicine, Department of Medical, Surgical and Experimental Sciences, University of Sassari, 07100 Sassari, Italy; angela.spanu@aousassari.it; 5 Unit of Pathology, Department of Medical, Surgical and Experimental Sciences, University of Sassari, 07100 Sassari, Italy; antonio.cossu@aousassari.it

**Keywords:** breast cancer, breast reconstruction, COVID-19, Regional nerve blocks, mastectomy, breast conserving surgery

## Abstract

The recent COVID-19 pandemic has caused profound changes to healthcare systems as well as had deleterious repercussions on the care of cancer patients. In this comparative study, we sought to evaluate the effects of the COVID-19 pandemic on the surgical management of breast cancer in a breast unit in an Italian region with a low incidence of COVID-19 infection. Eighty-three patients were included, of whom 41 received surgery during the height of the pandemic (Group A, operated on between March and April 2020), and 42 during the same period of the year in 2019 (Group B). Clinicopathological characteristics and surgical outcomes were compared between the two groups. There were no significant differences in the baseline characteristics of the two groups with regard to age (*p* = 0.62), tumour size (*p* = 0.25), grade (*p* = 0.27), histology (*p* = 0.43), positive lymph node status (*p* = 0.35), and ER positive status (0.35). Waiting time for surgery was slightly longer in Group A (49.11 vs. 46.39 days, *p* = 0.38). Patients receiving immediate breast reconstruction were significantly less in Group A (*p* < 0.001). The use of sentinel node biopsy was similar in the two groups (*p* = 0.84). Hospital stay was longer in patients of Group B (*p* = 0.008). The use of regional nerve blocks was lower in Group A (*p* < 0.001). Patients operated on during the height of the pandemic were less likely to receive immediate reconstruction and regional nerve blocks during surgery. These features configure a situation of reduced level of care for patients with breast cancer. Efforts should be taken by the healthcare systems to maintain standard of care, even in case of a new peak in the coronavirus outbreak.

## 1. Introduction

The COVID-19 is a recently discovered infectious disease caused by the SARS CoV-2 coronavirus, which was responsible for the current global outbreak of severe acute respiratory syndrome. Due to the highly infectious nature of COVID-19, the epidemic has rapidly spread since December 2019, and to date has caused more than 9,220,000 confirmed cases and almost 477,000 deaths worldwide [1]. 

The COVID-19 pandemic has led to radical transformations in the social and economic lives of individuals and communities. Above all, healthcare systems across the globe have witnessed profound changes, with tremendous effects on care of cancer patients. As a consequence, specific national regulations and guidelines have been adopted with the aim to mitigate the negative effects of the COVID-19 pandemic on cancer management [2,3,4,5].

Italy was the first country in Europe to be affected by the pandemic, and the one with the current highest number of deaths, although important differences in the burden of the outbreak were registered between the northern and southern regions, the latter having the lower case rate [6]. In particular, the island region of Sardinia has one of the lowest rates of infection and death from COVID-19, when compared to other Italian regions. Some of the reasons for this may be the insularity and travel restrictions and precautions put in place from the beginning of the epidemic in Italy. The measures taken to contain the spread of COVID-19 infection were adopted across the country, with no distinctions in differences related to rates of disease burden.

In this study, we sought to evaluate the effects of the COVID-19 pandemic on the surgical management of breast cancer patients in a breast unit in a region with limited COVID-19 cases, by comparing treatment and outcomes of patients operated on during the pandemic with those operated on in the pre-pandemic period.

## 2. Methods

This study was conducted at the Unit of General Surgery 2, University of Sassari (Italy). The unit is a part of the SMAC (Senologia Multidisciplinare Aziendale Coordinata—Italian acronym for Healthcare Company Coordinated Multidisciplinary Senology), which represents the referral center for breast cancer treatment in Northern Sardinia, and satisfies the requirements of a specialist breast center, as identified by the European Society of Mastology (EUSOMA) [7]. It is estimated that about 1400 new cases of breast cancer occur in the island of Sardinia every year, of which about 250 are operated on at the institution per year. For the purpose of this study, we identified two cohorts of patients. The first one was composed of patients operated on for breast cancer between 1 March 2020, and 30 April 2020, during the height of the epidemic (Group A). They were compared to a cohort of patients operated on for breast cancer during the same period in the year 2019, before the epidemic (Group B). The following variables were extrapolated for the entire study population: age, menopausal status, waiting time for surgery, tumour size, histological type, tumour grade, estrogen receptor (ER) status, Her2 status, axillary lymph nodes status, use of neoadjuvant chemotherapy, type of upfront surgery (BCS, mastectomy), use of sentinel node biopsy, use of immediate reconstruction, use of intraoperative regional nerve blocks, waiting time for postoperative oncological and radiotherapy consultation, and waiting time for chemotherapy. Waiting time for surgery was calculated as the number of days between the date of histological diagnosis of cancer and the date of surgery for patients receiving upfront surgery. For patients receiving neoadjuvant chemotherapy, it was calculated as the number of days between the last chemotherapy cycle administration and the date of surgery. Prior to entry into the hospital, all patients operated on in 2020 were triaged for possible symptoms of COVID-19, and had a nasopharyngeal COVID-19 test. None of them were found to be positive for the coronavirus infection. 

### Statistical Analysis

The patients’ demographic, tumour characteristics, and operative outcomes were summarized by using descriptive statistics. Continuous variables were evaluated as means ± standard deviations and compared using the t-test or the Mann-Whitney test, where appropriate. Differences in proportions between the two groups of the study were compared using the chi-square test. All *p* values lower than 0.05 were considered statistically significant. Statistical analysis was conducted by using SPSS Statistics 20 (IBM Corporation, Armonk, NY, USA).

## 3. Results

In the months of March and April 2020, 42 patients underwent surgery for breast cancer, in a period in which special measures were taken at the institution for the coronavirus outbreak (Group A). They were compared with 41 patients operated on during the same months in 2019 (Group B).

There were no significant differences in the baseline characteristics of the two groups in regard to mean age (*p* = 0.62), tumour size (*p* = 0.25), tumor grade (*p* = 0.27), tumour histology (*p* = 0.43), positive axillary lymph nodes (*p* = 0.35), ER positive status (0.35), Her2 positive status (*p* = 0.75), and use of neoadjuvant chemotherapy (*p* = 0.72) (Table 1). 

Globally, the mean waiting time for surgery was 47.8 days, and it was slightly longer in Group A (49.11 vs. 46.39), although the difference was not significant (*p* = 0.38). We found a difference in the type of surgical intervention performed (*p* < 0.001). In fact, in Group A, 32 (78.0%), patients received a BCS, 9 (22.0%) a mastectomy alone, and none of them received immediate reconstruction. In Group B, 29 (69.0%) had a BCS, 1 (2.4%) a mastectomy alone, and 12 (28.6%) a mastectomy plus immediate reconstruction. In regard to patient candidates for mastectomy, 12/13 (92.3%) patients from Group B were submitted to immediate reconstruction, vs. 0/9 in Group A. Use of sentinel node biopsy was similar in the two groups (78.0% vs. 76.2%, *p* = 0.84). Regional nerve blocks during surgery were performed more frequently in patients of Group B (90.5% vs. 2.4%, *p* < 0.002). Postoperative hospital stay was significantly shorter in patients of Group A (1.5 vs. 2.5, *p* = 0.008). Patients submitted to adjuvant chemotherapy were 6 (14.7%) and 11 (26.2%) in Group A and Group B, respectively. Endocrine therapy was administered to 35 (85.4%) patients in Group A and 28 patients (66.7%) in Group B. Waiting time for postoperative oncological consultation did not differ between the two study groups (24.9 vs. 23.1 days, *p* = 0.74), as well as waiting time for the commencement of adjuvant chemotherapy (42.8 vs. 45.4 days, *p* = 0.58). In addition, the waiting time for radiotherapy consultation did not differ (26.1 vs. 25.9, *p* = 0.77) (Table 2).

## 4. Discussion

The COVID-19 pandemic has had both immediate and delayed consequences on patients with breast cancer. We sought to evaluate the level of care for women operated on for breast cancer in an Italian region with a limited number of COVID-19 infections. As of 30 April 2020, a total of 205,463 confirmed cases were reported in Italy, including 27,967 deaths. Among them, only 1295 cases (accounting for 0.63 percent of all cases) and 116 deceased persons (accounting for 0.41 percent of all deaths) were registered in Sardinia. Nonetheless, in March and April 2020, the local hospital system was reshaped in order to meet the health burden in view of the rapid spread of the pandemic. Just as other authors have underscored, the coronavirus pandemic has prompted reorganization of hospital systems and patient pathways, mainly in the most affected countries [2,8,9]. Our university hospital has become a regional hub for the COVID-19 outbreak, where paramedical and medical staff, especially anesthesiologists and intensivists, have been reassigned to manage the rush of patients with the coronavirus infection. As a consequence, routine surgical activities have been reduced. In addition, several cases of COVID-19 infection were diagnosed among patients and health workers at the hospital between the end of February and the beginning of March; thus, we experienced a two-week suspension of elective surgical activities, which were partially diverted to other hospitals. 

The main results of our study are that during the two months when the pandemic reached its peak, we observed a significant reduction in immediate reconstruction after mastectomy, as well as a reduction in the use of regional nerve blocks during breast surgery. In addition, the waiting time for surgery was longer during the pandemic when compared to the pre-COVID era, although not in a significant manner. All these findings can be considered as an indicator of reduced level of care in patients undergoing surgery for breast cancer. 

The importance of immediate reconstruction in patients receiving mastectomy cannot be overemphasized. Several authors have underscored the advantages of immediate reconstruction in terms of psychological wellbeing and quality of life in general [10,11,12,13]. Moreover, recent researches have highlighted that contraindications to that procedure have progressively decreased. In a recent paper, we reported on the increasing rate of immediate reconstruction at our institution—12.6% in 2002 to 62.7% in 2016 [12]. In the present study, we observed a significant difference in the surgical treatment of breast cancer between the COVID-19 pandemic era and the same period in the pre-COVID era. In particular, no patient received immediate reconstruction during March and April 2020. This policy was consistent with similar policies adopted by other Italian Breast Units belonging to regions with the virus’ highest prevalence rates [14]. Patients were informed that the contemporary guidelines suggested to postpone non-urgent procedures and were reassured that delayed reconstruction would have to be scheduled according to the oncological situation and the decision of the multidisciplinary meeting [2,3]. Multidisciplinary meetings have continued during the pandemic in videoconference modality. In addition, psychological consultancies, which are part of preoperative work-up in our Breast Unit, have been offered as a video consultation service to all patients. The psychological concerns of patients who experience cancer treatment in the middle of a worldwide pandemic cannot be underestimated, and adequate psychological support should be provided. One of the main items in the management of breast cancer patients is to understand how reasonably, and for how long surgery can be delayed, taking into account that the highest quality of treatments should be maintained under any circumstance. As a matter of fact, the peak of epidemic emergency lasted for two months at the institution, and from May 2020, surgical activity returned to normal. Nevertheless, at the beginning of March, it was impossible to predict the actual duration and impact of the pandemic. This pushed us to ensure patients’ cure, however, to the detriment of certain important aspects, such as immediate reconstruction after mastectomy. Moreover, there are other reasons for postponing reconstructive breast procedures during the epidemic emergency. First, perioperative morbidity is higher and there is a not-negligible rate of reoperation for reconstruction-related complications. Second, reconstructive surgery usually requires longer postoperative stay, and this is not desirable in a time when hospital stay should be minimized [15]. As expected, in the present study, the hospital stay for patients operated on during the pandemic was shorter, mostly due to the avoidance of immediate reconstruction after mastectomy. Although we did not evaluate this data, postoperative outpatient visits were also likely reduced for the same reason, and this might have reduced the risk of COVID-19 infection in patients operated on for breast cancer. In this regard, it should be taken into account that studies have suggested that patients with cancer are a fragile population, namely more vulnerable to the new coronavirus infection [4,16,17]. To note, none of the women operated on for breast cancer in series presented herein resulted positive for the coronavirus infection.

In the present study, we found that peripheral regional nerve blocks were performed significantly less often in the pandemic period. Regional nerve blocks have a growing role in patients undergoing surgery for breast cancer. They are described to have some potential benefits, including better control of perioperative pain, faster recovery, early mobilization, and decreased occurrence of postoperative nausea and vomiting [18,19,20]. Moreover, they reduce the perioperative use of opioids. Although further evidence is needed, some studies have underscored that opioids commonly used for balanced anesthesia and postoperative pain control might negatively impact the survival outcomes in patients undergoing multimodality treatment for cancer [21,22]. Since the end of 2018, we have started to offer regional nerve blocks to all patient candidates for breast cancer surgery. In a recent report including 207 patients, we observed that regional blocks were associated to a reduced intraoperative use of opioids and analgesic drugs, and also to a reduced occurrence of postoperative nausea and vomiting. Moreover, we demonstrated that the selected patients can receive BCS with blocks plus sedation alone, avoiding general anesthesia [23]. However, we also found that the mean duration of surgery was slightly longer in patients who received regional blocks when compared with those who did not; thus, that practice was temporarily suspended with the aim to optimize operating times. Furthermore, some members of the anesthesiology service skilled in this particular technique were reassigned to the care of patients with COVID-19.

The rapid escalation of the COVID-19 outbreak has caused a global worsening of quality of care, which also involved breast cancer patients. It is of paramount importance to acknowledge that a new peak of the pandemic may occur in the near future, and healthcare systems cannot be unprepared for it, in order to maintain the highest quality standards of care to all patients. The possibility of obtaining immediate reconstruction during mastectomy, as well as having access to modern perioperative services, such as peripheral nerve blocks, represent an important part of quality of life for breast cancer patients. The recent recommendations of the European Society of Medical Oncology regarding the management and treatment of breast cancer in the COVID-19 era have classified ‘breast reconstruction with autologous tissue and/or implants’ as low priority [2]. This recommendation might also be a consequence of the preparedness of healthcare systems for rapid escalation of the pandemic, in a situation where one of the main efforts is to maintain a balance between the risk of contracting COVID-19 infection and control of the potential undesirable effects of cancer interventions. Thus, non-essential interventions such as breast reconstructions have been temporarily suspended. Nonetheless, it is our view that even in extraordinary situations, every patient should be able to receive high-quality cancer care, including interventions aiming to improve quality of life.

In a recent report evaluating elective surgery cancellations or postponements due to the COVID-19 pandemic during the peak 12 weeks of the outbreak, the COVID Surg Collaborative reported that the overall cancellation rate would be 37.7% for cancer surgery [24]. It has been suggested that the concept of “elective surgery” should be revisited in the current scenario. In fact, although breast cancer surgery may be considered a “nonemergency surgery”, the delay in tumour removal surgery may have detrimental effects not only on oncological outcomes, but also on quality of life. It has been reported that temporary suspension of elective surgery has had complex implications for patients with cancer and has also led to disparities in treatment [5,9,25]. Along with problems related to delay in treatment, an emerging issue is the delay in breast cancer diagnosis due to the COVID-19 pandemic. In a study from US including 278,778 patients, the authors reported a reduction by 51.8% of weekly number of patients with newly identified breast cancer during the pandemic, compared to the pre-COVID era [26].

In the present series, the waiting time for surgery was longer for breast cancer patients operated on in 2020, albeit not in a significant way. According to international and national guidelines, cancer surgery has been prioritized at the institution, although many teams have been reassigned to support other critical areas of the hospital. In particular, breast surgery usually does not require a postoperative ICU stay, unlike other types of oncological surgery. This probably has contributed to a non-significant delay in surgery for breast cancer patients in the present series. To note, waiting time for both postoperative oncology and radiotherapy consultations was not significantly increased among women operated on during the pandemic. Furthermore, no difference was observed in waiting time for the commencement of adjuvant chemotherapy. In cases in which the decision of the multidisciplinary team was in favor of endocrine therapy, the oncologic consultation was held by either videoconferencing or phone call. 

We acknowledge that this study has some limits, the main being its small sample size. However, to the best of our knowledge, it is one of the few studies addressing the impact of the COVID-19 outbreak on the quality of surgical care of patients with breast cancer in one of the countries with the highest incidence of pandemic. It also demonstrates that the COVID-19 pandemic may have detrimental consequences on the treatment of breast cancer patients, even in regions with a low incidence of COVID-19 infection. 

## 5. Conclusions

The COVID-19 outbreak may be associated with a reduced level of care for breast cancer. We observed that during the height of the pandemic, patients were less likely to receive immediate reconstruction after mastectomy and intraoperative regional nerve blocks. It is of paramount importance that healthcare services across the globe develop reliable and useful measures to maintain the highest standards of care in case of a new pandemic outbreak.

## Figures and Tables

**Table 1:** Demographic and clinicopathological characteristics of the study population.

Characteristic	Total (*n* = 83)	Group A—2020 (*n* = 42)	Group B—2019 (*n* = 41)	*p* Value
Age (mean ± SD)	61.3 ± 12.6	62.0 ± 12.4	60.6 ± 13.9	0.62
Tumour size (mean, SD)	17.7 ± 10.0	16.4 ± 9.2	18.9 ± 10.7	0.25
Tumour grade				0.27
I	15 (18.1%)	7 (17.1%)	8 (19.0%)	
II	48 (57.8%)	27 (65.8%)	21 (50.0%)	
III	20 (24.1%)	7 (17.1%)	13 (31.0%)	
Tumor histology				0.43
IDC	59 (71.2%)	30 (73.2%)	29 (69.0%)	
ILC	12 (14.4)	4 (9.7%)	8 (19.0%)	
DCIS	12 (14.4)	7 (17.1%)	5 (12.0)	
Positive Axillary lymph nodes	11 (13.25%)	4 (9.75%)	7 (16.6%)	0.35
ER positive	71 (85.5%)	37 (90.2%)	34 (80.9%)	0.35
Her2 positive	12 (14.4%)	5 (12.2%)	7 (16.7%)	0.75
Neoadjuvant chemotherapy	9 (10.8%)	5 (12.2%)	4 (9.5%)	0.72

DCIS: Ductal carcinoma in situ; IDC: Invasive ductal carcinoma; ILC: Invasive lobular carcinoma.

**Table 2:** Surgical and management outcomes of the study population.

Characteristic	Total (*n* = 83)	Group A—2020 (*n* = 41)	Group B—2019 (*n* = 42)	*p* Value
Waiting time for surgery	47.8 ± 12.6	49.1 ± 12.6	46.4 ± 11.6	0.38
Type of surgery				<0.001
BCS	61 (73.5%)	32 (78.0%)	29 (69.0%)	
Mastectomy alone	10 (12.0%)	9 (22.0%)	1 (2.4%)	
Mastectomy + IR	12 (14.5%)	0 (0%)	12 (28.6%)	
Sentinel node biopsy	64 (77.1%)	32 (78.0%)	32 (76.2%)	0.84
Regional nerve blocks	39 (47.0%)	1 (2.4%)	38 (90.5%)	<0.001
Hospital stay	2.0 ± 1.7	1.5 ± 1.02	2.5 ± 2.0	0.008
Waiting time for postoperative oncological consultation	24.0 ± 4.6	24.9 ± 4.6	23.1 ± 4.5	0.74
Waiting time for chemotherapy	44.5 ± 8.5	42.8 ± 10.1	45.4 ± 8.0	0.58
Waiting time for radiotherapy consultation	26.0 ± 3.4	26.1 ± 3.5	25.9 ± 3.4	0.77

BCS: Breast Conserving Surgery; IR: Immediate Reconstruction.

## References

[1] European Centre for Disease Prevention and Control. https://www.ecdc.europa.eu/en/geographical-distribution-2019-ncov-cases.

[2] De Azambuja E., Trapani D., Loibl S., Delaloge S., Senkus E., Criscitiello C., Poortman P., Gnant M., Di Cosimo S., Cortes J. (2020). ESMO Management and treatment adapted recommendations in the COVID-19 era: Breast Cancer. ESMO Open.

[3] Burki T.K. (2020). Cancer guidelines during the COVID-19 pandemic. Lancet Oncol..

[4] Nepogodiev D., Bhangu A., Glasbey J.C., Li E., Omar O.M., Simoes J.F., Abbott T.E., Alser O., Arnaud A.P., Bankhead-Kendall B.K. (2020). Mortality and pulmonary complications in patients undergoing surgery with perioperative SARS-CoV-2 infection: An international cohort study. Lancet.

[5] Schrag D., Hershman D.L., Basch E. (2020). Oncology practice during the COVID-19 pandemic. JAMA.

[6] Liotta G., Marazzi M.C., Orlando S., Palombi L. (2020). Is social connectedness a risk factor for the spreading of COVID-19 among older adults? The Italian paradox. PLoS ONE.

[7] Biganzoli L., Cardoso F., Beishon M., Cameron D., Cataliotti L., Coles C.E., Bolton R.C.D., Trill M.D., Erdem S., Fjell M. (2020). The requirements of a specialist breast centre. Breast.

[8] Vogler S.A., Lightner A.L. (2020). Rethinking how we care for our patients in a time of social distancing during the COVID-19 pandemic. Br. J. Surg..

[9] Balogun O.D., Bea V.J., Phillips E. (2020). Disparities in cancer outcomes due to COVID-19-A tale of 2 cities. JAMA Oncol..

[10] Fancellu A., Sanna V., Sedda M.L., DelRio D.M., Cottu P., Spanu A., Giuliani G., Conti M., Piras R., Crivelli P. (2019). Benefits of organized mammographic screening programs in women aged 50 to 69 years: A surgical perspective. Clin. Breast Cancer.

[11] Albornoz C.R., Cordeiro P.G., Farias-Eisner G., Mehrara B.J., Pusic A.L., McCarthy C.M., Disa J.J., Hudis C.A., Matros E. (2014). Diminishing relative contraindications for immediate breast reconstruction. Plast. Reconstr. Surg..

[12] Fancellu A., Sanna V., Cottu P., Feo C.F., Scanu A.M., Farina G., Bulla A., Spanu A., Paliogiannis P., Porcu A. (2018). Mastectomy patterns, but not rates, are changing in the treatment of early breast cancer. Experience of a single European institution on 2315 consecutive patients. Breast.

[13] Siotos C., Lagiou P., Cheah M.A., Bello R.J., Orfanos P., Payne R.M., Broderick K.P., Aliu O., Habibi M., Cooney C.M. (2020). Determinants of receiving immediate breast reconstruction: An analysis of patient characteristics at a tertiary care center in the US. Surg. Oncol..

[14] Corsi F., Caruso A., Albasini S., Bossi D., Polizzi A., Msc F.P., Truffi M. (2020). Management of breast cancer in an EUSOMA-accredited Breast Unit in Lombardy, Italy, during the COVID-19 pandemic. Breast J..

[15] Di Pace B., Benson J.R., Malata C.M. (2020). Breast reconstruction and the COVID-19 pandemic: A viewpoint. J. Plast. Reconstr. Aesthetic Surg..

[16] Disis M.L. (2020). Oncology and COVID-19. JAMA.

[17] Liang W., Guan W., Chen R., Wang W., Li J., Xu K., Li C., Ai Q., Lu W., Liang H. (2020). Cancer patients in SARS-CoV-2 infection: A nationwide analysis in China. Lancet Oncol..

[18] Choi J.J., Jo Y.Y., Shin Y., Jung W.S., Lee D., Kim K.Y., Kwak H.J. (2019). Remifentanil-sparing effect of pectoral nerve block type ii in breast surgery under surgical pleth index-guided analgesia during total intravenous anesthesia. J. Clin. Med..

[19] Garg R., Bhan S., Vig S. (2018). Newer regional analgesia interventions (fascial plane blocks) for breast surgeries: Review of literature. Indian J. Anaesth..

[20] Singh P.M., Borle A., Kaur M., Trikha A., Sinha A. (2018). Opioid-sparing effects of the thoracic interfascial plane blocks: A meta-analysis of randomized controlled trials. Saudi J. Anaesth..

[21] Exadaktylos M.A.K., Buggy D.J., Moriarty F.D.C., Mascha P.E., Sessler M.D.I. (2006). Can anesthetic technique for primary breast cancer surgery affect recurrence or metastasis?. Anesthesiology.

[22] Forget P., Vandenhende J., Berliere M., Machiels J.-P., Nussbaum B., Legrand C., De Kock M. (2010). Do Intraoperative analgesics influence breast cancer recurrence after mastectomy? A retrospective analysis. Anesth. Analg..

[23] Fancellu A., Perra T., Ninniri C., Cottu P., Deiana G., Feo C.F., Porcu A. (2020). The emerging role of pectoral nerve block (PECS block) in breast surgery: A case-matched analysis. Breast J..

[24] Nepogodiev D., Bhangu A., COVID Surg Collaborative (2020). Elective surgery cancellations due to the COVID-19 pandemic: Global predictive modelling to inform surgical recovery plans. Br. J. Surg..

[25] Meredith J.W., High K.P., Freischlag J.A. (2020). Preserving elective surgeries in the COVID-19 pandemic and the future. JAMA.

[26] Kaufman H.W., Chen Z., Niles J., Fesko Y. (2020). Changes in the number of US patients with newly identified cancer before and during the coronavirus disease 2019 (COVID-19) pandemic. JAMA Netw. Open.

